# Interaction of Secure Cloud Network and Crowd Computing for Smart City Data Obfuscation

**DOI:** 10.3390/s22197169

**Published:** 2022-09-21

**Authors:** Manikandan Thirumalaisamy, Shajahan Basheer, Shitharth Selvarajan, Sara A. Althubiti, Fayadh Alenezi, Gautam Srivastava, Jerry Chun-Wei Lin

**Affiliations:** 1 School of Computing Science and Engineering, Galgotias University, Greater Noida 203201, India; 2 Department of Computer Science, Kebri Dehar University, Kebri Dehar P.O. Box 250, Ethiopia; 3 Department of Computer Science, College of Computer and Information Sciences, Majmaah University, Al-Majmaah 11952, Saudi Arabia; 4 Department of Electrical Engineering, College of Engineering, Jouf University, Sakaka 72388, Saudi Arabia; 5 Department of Mathematics and Computer Science, Brandon University, Brandon, MB R7A 6A9, Canada; 6 Research Center for Interneural Computing, China Medical University, Taichung 40402, Taiwan; 7 Department of Computer Science and Mathematics, Lebanese American University, Beirut 1102, Lebanon; 8 Department of Computer Science, Electrical Engineering and Mathematical Sciences, Western Norway, University of Applied Sciences, 5063 Bergen, Norway

**Keywords:** AODE classifier, cloud computing separation, data obfuscation, data storing, data transmission, data classification

## Abstract

There can be many inherent issues in the process of managing cloud infrastructure and the platform of the cloud. The platform of the cloud manages cloud software and legality issues in making contracts. The platform also handles the process of managing cloud software services and legal contract-based segmentation. In this paper, we tackle these issues directly with some feasible solutions. For these constraints, the Averaged One-Dependence Estimators (AODE) classifier and the SELECT Applicable Only to Parallel Server (SELECT-APSL ASA) method are proposed to separate the data related to the place. ASA is made up of the AODE and SELECT Applicable Only to Parallel Server. The AODE classifier is used to separate the data from smart city data based on the hybrid data obfuscation technique. The data from the hybrid data obfuscation technique manages 50% of the raw data, and 50% of hospital data is masked using the proposed transmission. The analysis of energy consumption before the cryptosystem shows the total packet delivered by about 71.66% compared with existing algorithms. The analysis of energy consumption after cryptosystem assumption shows 47.34% consumption, compared to existing state-of-the-art algorithms. The average energy consumption before data obfuscation decreased by 2.47%, and the average energy consumption after data obfuscation was reduced by 9.90%. The analysis of the makespan time before data obfuscation decreased by 33.71%. Compared to existing state-of-the-art algorithms, the study of makespan time after data obfuscation decreased by 1.3%. These impressive results show the strength of our methodology.

## 1. Introduction

This study focuses on the upkeep of Internet of Things (IoT) sensor data in smart sensor cities and its management through cloud computing. Cloud computing is the process of storing, managing, and accessing data through the Internet as opposed to on a local server or personal computer (PC). Cloud computing is becoming more popular and advantageous for individuals, companies, and organizations. The processing of physical devices in IoT allows data to be shared from one device to another. To prevent work overload, work is shared from one device to another. Organizations can use IoT to examine how their systems work in real-time and obtain insights into everything from equipment performance to supply chain and logistics operations. The IoT work-sharing technique controls the system’s performance lagging [1]. IoT is suggested for the collection of sensor data from multiple crowdsourcing datasets. These links are built on the smart cities that use sensors in hospitals, on the sides of roadways, and in virtual environments for crowd interactions using cloud technology [2]. The categorized AODE is used to classify the data. This organizes the crowd data gathered from various sensors and smart cities. The majority of hospitals and roadside places are categorized in the data gathered from smart cities. The AODE in sensor data is used to categorize this data collection and assign it to different data storage. This storage completes the necessary database, which controls the information needed to reach the suggested conclusion. Additionally, a method for storing data behaviour and redundancy has been suggested. The sensor-based data has advanced, and this controls how cities are formed. Hybrid data obfuscation is used to manage the method of classifying the data for the blockchain facilities [3]. The security objective for data management and data formation is made for security purposes by data obfuscation [4]. The management of data encryption masking and data tokenization for the original data is handled by data transfer from smart cities. Environmental production controls the process, and an external processor is suggested for moving records from one location to another. Data encryption is managed by data security to handle original data for production environments on both public and private systems. Personal data has been used for a different type of data obfuscation function [5]. The system structure for the cryptosystem is used to manage the encrypted data. This security, which evolves through the creation of additional storage for each connected piece of data, can be managed by the database. Text, photos, GIFs, and videos are the categories used to categorize the factors for storing data based on associated images. The hybrid data obfuscation technique is also used for this classification’s data storing process [6]. When using cloud-based services, this research depends on remote servers for our technological infrastructure. Since cloud computing encourages mobile access via smart devices, it keeps people informed. It decreased IT expenditures. If you move to the cloud, the cost of managing and maintaining your IT systems may can thus go down. Scalability, company continuity, collaborative effectiveness, flexibility in work procedures, and accessibility to automated upgrades are other advantages that can be accessed remotely from the cloud, or another virtual location is likewise covered by this. Thanks to companies that provide cloud services, users can store files and apps on remote servers and then access the material via the internet. The synergy of cloud and crowd computing for the smart city focused on maintaining the data collected from the IoT sensor in smart sensor cities and managing it in cloud computing. The data obfuscation, classification, and crowd computing methods are performing cloud computing platforms for improving the data storing and retrieving which is got from smart cities using sensors. The data collection and classification using the crowd computing approach are emphasized. Data collection uses the IoT sensor for storing big cloud data. In the approach of crowd computing, the separation of the data from the smart cities is collected and separated using the technique of AODE classifier; the classification is done (collection of data from hospitals, stations, etc.) The cryptosystem and data obfuscation has hybrid data obfuscation technique). For improved data security, hybrid data obfuscation is used for transmitting from while storing in the big data. For data retrieving, the SELECT-APSL method from the cloud is based on the data from the IoT sensor from smart cities and hybrid data obfuscation for security purposes. The SELECT-APSL method was used in this paper to retrieve the data. The single-column in-pages dexes are used to obtain the data from the table. Only the index page is used for retrieval during the procedure. This page serves as the primary scanning point for both the multi-column index scan key retrieval and the data retrieval for the plugin [7]. This retrieval of the data from the smart cities with the security details has been done. The main objective of the paper is as follows:

- The data collected from the smart cities using the crowd to the cloud computing is proposed by using the AODE classifier.
- The hybrid data obfuscation technique has been used for security purposes for data analysis.
- Also, for retrieving the data, the method of SELECT-APSL is saved in the cloud.

The rest of the paper is organized in Section 1, the introduction. In Section 2, the related works, and in Section 3, the proposed system is presented. In Section 4, the methods and the results have been elaborated in graphs and tables. In Section 5, the conclusion has been written along with future work.

## 2. Literature Review

Different layers of firmware and middleware have performed sophisticated services for real-world and cyber-physical combinations for CPS in this study. This makes cloud computing possible and controls the latency caused by geographic distance for the user’s orchestration of services and resources. These cloud-based data have been gathered from industry 4.0, smart households, and smart cities. This can be integrated into the currently suggested architecture to enable cloud and crowd computing in a federated environment [8]. In this study, a crowd-based SciCrowd system is introduced, and a scholarly literature search is developed for various research communities. These studies include data indexing, automatic paper extraction, and crowdsourced data processing. This allows for data searching based on intelligent cities in the currently planned system [9]. The decision-making process and land management for planning for repercussions are done for new opportunities in this manuscript, which controls social sensing data. A geospatial data algorithm for the analysis of complex data for land governance is suggested. By incorporating this technique into the system that is currently being presented, social goals to manage energy extraction can be suggested [10]. The fifth generation of drones is proposed as edge intelligence for controlling artificial intelligence. To construct intelligent settings, security based on data sharing and applications based on blockchain is used. Additionally, obstacles for drone edge intelligence have been created to manage upcoming data-sharing tendencies. This can be done in the federated synergy system that is currently being suggested [7]. It has been suggested that autonomous aerial vehicles and a revolutionary framework would enable future smart cities. Additionally, the automated necessity of an intelligent ecosystem is recommended in the process of locating autonomous cars and decision-making among smart cities and light plains. Additionally, a mechanism for automating FAUAVs has been suggested. Swarm intelligence has been presented to manage the different missions with this rising efficiency. By managing this in the currently suggested system, it has been suggested that unnamed aerial vehicles be controlled and monitored [2]. Future trends data and surveillance of intelligent cities have been examined in this research based on the data collection. Additionally, an embedded system for visual computing and future trends and problems, as well as video surveillance for the most recent data set, are studied. The analysis of data based on big data has been evaluated by incorporating this into the system that is now being suggested. This article was motivated by technical elements for the development of the economy and blockchain based on the secrecy of flaws and constructing a system that improves vital component qualities [11]. Effective authentication and donation implementation have been suggested. The construction of a useful smoothing of blockchain security is managed in this work. Based on concerns in current developments in technology-based cities and IoT smart cities, Mukherjee et al. suggest intelligent cities by integrating this into the present system and controlling academic references of cloud-based data, IoT-based intelligent administration, factors for innovative entities, and crucial decisions based on the smart city are offered [12]. This motivated the author to participate in the currently suggested system by retrieving data from a database [13]. To increase the deep and broad correlations of different channels and produce a successful SR network, Tian et al. introduced a 40-layer ESRGCNN [14]. This study uses adaptive up-sampling to create a flexible SR model that is especially useful for real-world uses. This study concludes that, in addition to the shallow ESRGCNN used in the number of parameters to layer RDN and CSFM for obtaining excellent visual effects, the super-resolution group CNN (ESRGCNN) for SISR will enhance the effect of deep and wide channel features by correlations of different channels to ESRGCNN for inheriting. It works with low-resolution images captured using different sampling strategies. This study achieves Comprehensive SISR objectives in terms of complexity and visual quality.

Finally, this literature review concentrated on the firmware and middleware layers concerning cyber-physical cloud computing. By doing this, the geographical delay is reduced. The SciCrowd crowd-based technology that has been presented develops academic literature search for various research communities. Multiple variables and planetary-scale geographical data were considered in the decision-making process [15]. Data exchange, analysis, and decision-making are the foundations for security when it comes to autonomous aerial aircraft [16], detecting autonomous vehicles, and making decisions. The effectiveness of monitoring unidentified cars has been improved by several existing automation systems. The most recent data for embedded systems is applied to the video surveillance system. Big data-based information provides motivating guidelines for advancing the economy and blockchain privacy. These studies provide an interesting idea regarding blockchain security.

## 3. Proposed Smart City Data Acquisition

The use of (ICTs) to improve quality of life, efficiency, and competitiveness, while ensuring that it meets the needs of present and future generations it represents Smart buildings, smart vehicles and roads, smart energy management, smart home, and so on [12]. A smart city is an intelligent city that is integrated digital technologies into its networks, services, and infrastructures. A smart city is a municipality that uses information and communication technologies to increase operational efficiency, share information with the public, and improve the quality of government services and citizen welfare.

Data collection is one of the critical building blocks of smart city applications. It uses Internet of Things (IoT) devices such as sensors, meters, among others, to collect and analyze data. The data in smart cities helps to improve infrastructures, public utilities, and services and to manage the daily task of enhancing public safety and environmental issues [17]. Here, data are collected from multiple sources. These sources are everywhere, including environmental sensors, cameras, GPS, smart gadgets, etc. Several big data can be arranged and stored at various sites for analysis. Many sensors, cameras, and other devices are utilized for data collection as the different types of data from different sources are required for analysis and to provide resources [10]. The entire process takes place with the help of the internet.

These data are generated continuously every second and every day, so the data are too large and complex to manage and store in a local server. In addition, this is not secure for data. Hence cloud computing technology is used for data storage and process. Cloud computing can store and manage a massive volume of data with the functionality of scalable and virtualized resources for computation by integrating resources of the electrical power system through a network. This increases the capacity of storage, robustness, and load balancing. It provides resources to users anytime and anywhere whenever a resource is required [11].

**Figure 1:**
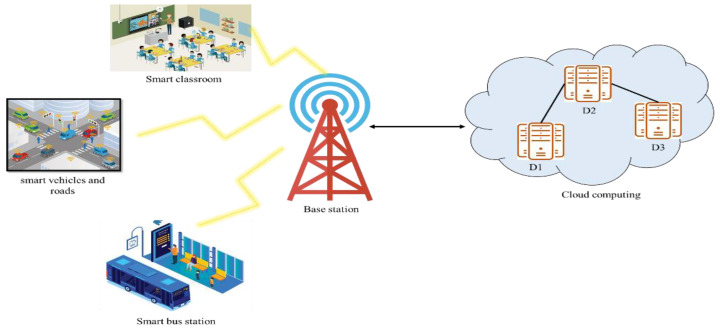
Data collection and storage in smart cities.

The smart city is based on the development of technology in the environment and society to make life easier and more comfortable, i.e., smart classrooms, smart vehicles, smart bus stations, and many others. The data collection is through devices, sensors, cameras, and so on with the help of the Internet [17]. Sensors are installed in indoor and outdoor cities, which provide the data by observing the signals that support and measure the various types of data from multiple places that can be converted to give understandable data. These collected data are stored in the cloud for future access or analysis, as shown in Figure 1 [18].

The sensors monitor the smart cities’ environment that rates the acceptable range of data. Each sensor holds an AODE classifier for the multiclassification purpose of each sensor collected data. This AODE (Averaged One-Dependence Estimator) classifier is a supervised machine learning algorithm that necessitates the attributes dependent on one another by averaging the whole classifier [15]. The AODE classifier classifies the collected data as normal or abnormal environment data by considering the majority voting. Algorithm 1 shows the steps for collecting data from IoT sensors in Smart Cities. 

**Algorithm 1:** Algorithm for Collecting Data from IoT Sensors in Smart Cities.*Begin*  *Input: A= {a1…an}-instance, R- result  // training data* *For each instance*  *Initially, the sensor finds a neighbour sensor, NN*  *p (b, a) = p (b| a) p (a)*     $\;P(b/a)=\frac{p\left(b\right)p(a|b)}{p\left(a\right)}$*  //Compute the probability of b given a:* *The end for // testing data* *Repeat* *For ak ϵ A*    *Compute p (b, a) = p (b, ak) p (a| b, ak)*     
$\;P(b/a)=\frac{p\left(b,a_{k}\right)p(a|b,\;a_{k})}{p\left(a\right)}$
 *Get R☐ node NN* *Return class value* *Update classifier* *End for* *Until Convergence* *End.*


For instance, each sensor held the AODE classifier. One of the sensors acts as a global classifier to predict the final output that collects the data from the local classifier. For each *a_k_* belongs to the *A*, and AODE looks for an estimate of the probability of each class *b* as follows,

$$P(b/a)=\frac{p\left(b,a_{k}\right)p(a|b,a_{k})}{p\left(a\right)},$$

where *P(b/a)* represents the estimation of *p*$\left(b,a_{k}\right)$. The input sample data and *n* count of sensors. The prediction is based on the network traffic as either normal or abnormal data using distributed majority voting [19]. Consider an odd sensor count for distributed majority voting when the primary or highest vote determines the network traffic pattern. Finally, based on the sensor count, we can find the probability of data as normal or abnormal.

### 3.1. Crowd Computing in Smart City

The crowd represents a collection or group. It is a distributed computing model where a huge non-trivial process or task is split into several independent atomic or individual tasks distributed over multiple computing devices for each process [20]. Similarly, in crowd computing, the crowd determines a group sharing the resources or an ideal CPU cycle of a device for doing different computational processes. As many tasks are continuously taking place, it becomes difficult to manage them, so crowd computing is adapted [2].

These individual tasks are represented as micro-tasks, always in a ready state inside a job pool. Then the available crowd workers are searched, and a set of required crowd workers are selected. These crowd workers are potential to provide flexible support to overcome the challenges in managing and allocating work properly by assigning each task from the job pool to a variety of selected crowd workers and, in some cases, to maintain reliability, the same tasks might be given to different workers [18]. These individual tasks are provided as a simple program to the crowd workers without any other information depending on the context. Each crowd of workers produces a separate report of output to the centralized master, where all the results of each task are gathered after the execution of an independent individual task. After collecting those outputs, it masters checks for errors and assembles them to the final result set [21].

The development of technologies such as the Internet of Things (IoT), Artificial Intelligence (AI), cloud, and other technologies paved the way for the growth of smart cities worldwide [22]. It is a promising technology with the functionality of using computing resources in a scalable and virtualized manner [20]. The city is called smart when the other fields such as transportation, public utilizes, education, smart home, public safety, and social and health care become smart, so it is found there is extensive use of data; thus, to manage these data, a cloud with crowd computing is used in this system [23]. 

**Figure 2:**
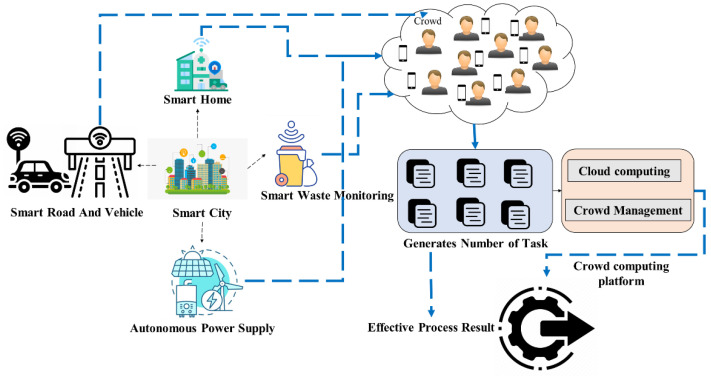
Crowd Computing with the Cloud for a smart city.

As the single field contains the extensive data collected and managed by cloud computing, different areas need to be gathered, maintained, monitored, and continuously accessed in the smart city. It becomes difficult to apply crowd computing, which works as a distributed system with complex tasks that are hard for computers and are handled by distributing the task across the Internet [13]. The extracted set of tasks is managed by crowd management which contains a steady flow of crowds to prevent large crowds and ensure that the tasks are controlled in an orderly manner for accessing the resource from the cloud, which produces effective processing results—shown in Figure 2.

### 3.2. AODE-Based Classification

In this system, an AODE method is used to separate data gathered from smart cities, such as traffic data, power supply data, etc. These data should be managed individually to provide resources effectively. AODE represents Averaged One-Dependence Estimator; it is a method of classification learning which forms a specific format of a Bayesian classifier network named single dependence classifier. This method allows mutual dependencies between value pairs within a vector of input when ignoring complicated relationships of dependencies that take place in three or more values to remove some of the naivety of naive Bayesian classifiers [16].

It performs well with a considerable number of training or input data. AODE performs classification by aggregating the predictions of all single dependency classifiers in which all attributes depend on the same single parent attribute and the class. That parent attribute satisfies a minimum frequency constraint. It is an effective method for accommodating the violations of independence attributes of Naïve Bayes to allow dependent attributes from other attributes of non-classes [24]. To maintain efficiency, it uses a single dependency classifier such as TAN, where each attribute is based on the class and, at most, one other attribute. The learning process in a single dependency classifier is conducted through model selection, which is a process that generally uses substantial computational overheads and increases the number of variances related to Naïve Bayes. However, AODE considered averaging the predictions of a single-dependency classifier to avoid the model selection process [5].

In every dependency classifier, the attributes are considered as a parent of all other attributes, and these attributes are known as super parents. This type of classifier is known as a super parent single dependency classifier; that is, only those classifiers with a value ai which takes place at least *n* times, were used for predicting a class label *b* for the instance of test a=a1, a2, …,ai thus for any values of attribute ai is represented as,

(1) $$P\left(b,\;A\right)=P\left(b,\;ai\right)\;P(A|\;b,ai)$$

as equality remains for every *ai*. Therefore, it becomes

(2) $$P\left(b,\;A\right)=P\;\left(y,x\right)\frac{\sum_{1\leq i\leq n\wedge F\;\left(x_{i}\right)\geq m}^{}P\;\left(y,x_{i}\right)P\;(x|y.x_{i})}{\left|\left\{1\leq i\leq n\;\wedge \;F\left(x_{i}\right)\geq m\right\}\right|}$$

where *f*(*ai*) determines the attribute value in frequency *ai* in the training sample dataset. Considering Equation (1), assuming that the attributes are independent, providing the class and super-parent *ai*. Now, AODE predicts the class for *A* by choosing,

(3) $$\ddot{Max\overset{1}{\underset{1\leq i\leq n\;\;\wedge \;\;\;F\left(x_{i}\right)\geq m\;}{\sum }}\;\hat{P}\;\left(y,x_{i}\right).\;\;\underset{1<j<n\;\;\wedge \;\;\;j\neq i\;}{\prod }\hat{P}\;\left(y,x_{i}\right)\;}$$

After that, each dependency classifier estimates the joint probability *P*(*b*, *A*). AODE is used to average the many estimates of a single term since it lowers the estimates’ variance. It includes significantly less bias by raising variance in a narrow range because AODE provides a weaker attribute that is a conditional independence assumption than Naive Bayes during the avoidance of the model selection process [13].

Numerous studies claim that it consistently has a much lower loss—0 to 1—than a naive Bayes technique utilizing a medium-time complexity. As a result, in those studies, an AODE has a mathematically significant advantage of 0 to 1 loss over many other semi-naive Bayesian algorithms. It offers classification accuracy comparable to state-of-the-art methods’ discrimination accuracy. AODE must update the estimated probability whenever a new instance becomes easily accessible. Because of this, incremental learning techniques also leverage this mechanism. Thus, AODE has much promise and is utilized as a replacement for a classification approach due to its many attractive qualities [25].

### 3.3. Hybrid Data Obfuscation Technique

**Figure 3:**
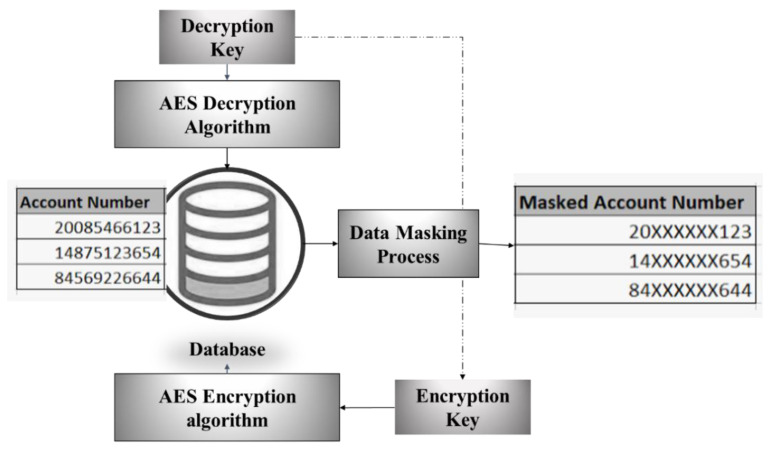
Hybrid data obfuscation for security.

Data security is critical in the Cloud storage method, as it provides a large amount of storage medium to store data collected from smart cities and other sources. These data might be accessed illegally without proper authentication using a piece of code; thus, it should be encrypted or masked for security to protect data from unauthorized access [9]. However, in this system, to strengthen data security [26,27,28], both the encrypted and made formats are used, i.e., cryptosystem and data obfuscation methods are used, and its combination is known as Hybrid data obfuscation; this is shown in Figure 3.

#### Secure Data Handling by Data Obfuscation

Data obfuscation is a masking format that alters sensitive data in such a manner that it is of little or no use to unauthorized intruders but is usable by a legitimate individual. The data masking is used to safeguard sensor data that is saved in the cloud. This information might be sensitive in terms of commerce, health, etc. Data masking replaces the actual data with plausible but bogus data to protect privacy. Any data masking or obfuscation should not change the meaning of the data; instead, the data must be sufficiently altered such that it is not immediately apparent that the masked data came from a source of production data [7].

Data obfuscation may be done using a variety of methods. We employ methods of replacement in this system. Substitution is one of the best ways to maintain the actual appearance and feel of the data records. This method is most effective in masking the overall data subset is a masked data set for several different data fields. Substitution masks the original value by replacing the data with a different value. This one is one of the best data masking techniques that keeps the data’s original appearance and feel. Several different sorts of data may be used using this method. By applying anonymity to the data records and mixing the data with the data structure, it is, therefore, simple to keep the data using this technique while simultaneously maintaining a realistic-looking database that is difficult to distinguish from a database made up of masked data [25].

While maintaining the services in the cloud, the hybrid data obfuscation technique (obfuscation and cryptography) is a more powerful tool that secures the data from a malicious user. When the user uses the cloud service to perform the task, the user needs to obfuscate the encrypted data without knowing anyone about the sensitive data. This leads the cloud server to finish the task without losing data privacy [29].

In the cryptographic method, as data encryption is used, the symmetric and private keys are used to encrypt the sensor data into unusable form till these data are decrypted. When encrypting the data, no one can analyze the data. Here we use the public-key cryptographic method for encrypting and decrypting the data [5]. The public key can be shared with anyone, while the private key is protected. Compounding the private and public keys, we can unlock the data file. Here we test the numerical sensor data in a cloud array using a hybrid data obfuscation technique. Algorithm 2 shows the hybrid data obfuscation process for security purposes. 

**Algorithm 2:** Algorithm for Hybrid Data Obfuscation for Security Purposes.
***Input:***
*P_t_- plaintext, C_T ☐cipher Text*

***Output:***
*data is Obfuscated*

***Initially***
*, P_t_- plaintext with n size*

*Get k_1_*

*** For each***
*k, j = 1,2… <=m*

***   find***
*square (Sq)*

   N_t_(k) = P_t_
(k)*k_1_        // value N_t_(k)

*   Sq(k) = pow (N_t_(k),2)     //Rotate the Sq(k)*

*   Get k_2_           //Rotate the RTN at K2 several times*

*   R_t_(k) = rotate (Sq(k), k_2_+j) //Find mod for RTN by 256*

*   mod(k) = R_t_(k)%256   //Convert mod into ASCII code*

* C_T (k) ☐ASCII (mod(k))*

* C_T☐ cipher text*

*End for*


Using these two keys, the hybrid data obfuscation technique conceives the numerical data to ensure data in the cloud server. These techniques use (mul), (pow), (pivot), (mod), and (ASCII) as coherent activities for data. In the cloud, private keys are created and sent to the users [3]. It retains the service in the company’s Key Management (*K_m_*). Initially, consider the sensor resulting from numerical data as plaintext with the size of the plain text. Then, the fair value of an *Sq*(*k*) and *N_t_*(*k*) = are computed. The given plaintext is multiplied with the *k*_1_ model calculation and stored as *N_t_*. Then calculate a value for *k*_1_, assign a value equal to the *m*, and multiply plaintext with the *k*_1_.

*N_t_*(*k*) = *P_t_* (*k*)∗*k*_1_ (4)

The model *k*_2_ is used for the qualities of the square. For *k*_2_ times, the *k*_2_ route also increased, and the fair value is rotated from right to left as Rotate the *Sq*(*k*). for upcoming values in *Sq*(*k*), *k*_2_ + *j*, where *j*, *k* = 1,2, 3... *N*.

*R_t_*(*k*) = rotate (*Sq*(*k*), *k*_2_ + *j*) (5)

Every time, attributes are released to clear for *k*_2_ by adding the mod value. Find *R_t_*(*k*) mod by 256. *R_t_*(*k*) mod value is computed by dividing the rotate value by 256. Every mod-value the Creating an ASCII character. The original numerical plaintext and ciphertext are represented by those ASCII letters. To create the ciphertext C T, convert *mod*(*k*) into ASCII code. A way to encrypt data without compromising its privacy is via cipher text [2]. The obfuscation creates the ciphertext by blending a variety of ASCII character codes. Each numerical value has a unique cipher text based on the ASCII characteristics code. Each character in the cipher text has an identical plaintext. The plain text and CipherText data sizes may differ [30,31,32].

### 3.4. Proposed Cryptosystem for Data Management 

A cryptosystem uses cryptographic methods and the infrastructure that supports them to offer information security services. A cipher system is another name for a cryptosystem. Plaintext, ciphertext, decryption key, and encryption key are the different parts of a fundamental cryptosystem. The data are encrypted using plaintext during the ciphertext storage procedure. When retrieved, the decryption technique is used to restore the original data [33,34,35,36]. Symmetric Key Encryption and Asymmetric Key Encryption are the two categories of encryption-decryption-based cryptosystems [20]. Asymmetric Key Encryption is the name given to the encryption technique when distinct keys are used to both encrypt and decode the data. It is possible to recover the plaintext by decrypting the ciphertext even though the keys are distinct yet mathematically linked. It makes use of separate private and public keys. For encryption and decryption, public and private keys are utilized [6]. The private key is meant to be private, as suggested by the name, so that only the receiver who has been verified may decode the message. To create a key pair, this technique employs a key generation protocol. Mathematically speaking, both keys are related. The way the keys are related varies depending on the algorithm [17]. Asymmetric encryption has benefits when storing data since it uses a public key that is widely available to encrypt data and a private key that is linked to a public key to decode data using the appropriate algorithms. Additionally, it includes the Parallel Server system’s SELECT-APSL command [29].

#### SELECT-APSL

SELECT-APSL: this is only applicable to Parallel Servers. The best join technique could be determined by the value of the? Parameter while the criteria are given with one. The value of the? The parameter cannot be known during pre-processing. Hence the best join technique cannot be chosen. The hit rate during SQL execution is calculated to select a joining technique [17]. The SELECT-APSL data retrieval method obtains the obfuscated hybrid data from a big-data database in a cloud server. The SELECT-APSL is only applicable for the Parallel Server (PS), which contains the results of obfuscated hybrid data. We consider the input as pre-processed data (PP_d_) and read the input of the Parallel Server as PS_in_. This input is selected and retrieved using a SELECT-APSL method [6]. Algorithm 3 shows the data retrieval process using SELECT-ASPL.

**Algorithm 3:** Algorithm for Data Retrieval Using SELECT-ASPL.***Initially**, the Parallel Server (PS) contains obfuscated hybrid data(O-H_d_), PP_d_- pre-processed data, input (Parallel Server)- PS_in_* ***Input:****PS_in_, PP_d_* ***For****each input* * Select ☐input (PS_in_)  // find PS_in_ using APSL* ***If****(specified big data DB = O-H_d_)* *select ☐ (Join method. (O-H_d_))* * O(O-H_d_)☐( O-H_d_)* ***While****pre-processing* * pre-processing_data count ≠ select optimum join method* ***return**, specified big data DB ≠ O-H_d_* ***End while*** ***End if*** ***If** (PP_d_ ≠ (O-H^d^)) // Compute hit rate* ***If** (hit rate data count ≥ no. of O(O-H_d_) + no. of PP_d_)* *** Else**     // data retrieving rate positively high* *check no. of PP_d_* ***End if End if*** ***Repeat*** ***If** no. of PP_d_> no. of (PS_in_)*   *execute: Compute the hit rate* ***Else if** (no. of PP_d_ < no. of (PS_in_)*   *continue (data retrieving rate positively low)* ***Else** rechecks no. of PP_d_* ***End if** (**Until** termination criterion met)* ***End for*** ***Return** accurate data retrieving rate && retrieve data as PSin*

This retrieval method only obtains the data when the specified condition is satisfied. When the specified big-data database containing the obfuscated hybrid data condition is valid, we can select and perform a join method that depends on the count of obfuscated hybrid data. Then we can optimize the obfuscated hybrid data and obtain an optimum join method. During the pre-processing process, we cannot determine the count of obfuscated hybrid data, so the specified big-data database does not contain the obfuscated hybrid data. When the pre-processed data is not similar to the obfuscated hybrid data, we select the join method for computing the hit rate. This computation leads to the rate of the data retrieving process [13].

When the count of hit rate is more significant than equal to the addition of the count of optimized [37] obfuscated hybrid data and pre-processed data, the possibility of data retrieving rate increases positively; otherwise, we need to check the count of pre-processed data [18]. When the count of pre-processed data is greater than the count of the parallel server, then we re-execute the computation process of hit rate. If the count of pre-processed data is lesser than the count of the parallel server, the time taken to retrieve the data rate is positively low. Otherwise, we recheck the count of pre-processed data [25].

The above process is repeated until we retrieve the final output as a parallel server input. This process finally retrieves the accurate hit rate as retrieving rate. We can easily retrieve the required data as an output [12]. At first, data is masked using a supplementation method. Then an asymmetric key is used to encrypt data using a private key [19]. This makes data more secure [38,39]. Thus, hybrid data obfuscation uses a cryptography system and obfuscation.

## 4. Result Comparison Discussion with Data Modules

**Figure 4:**
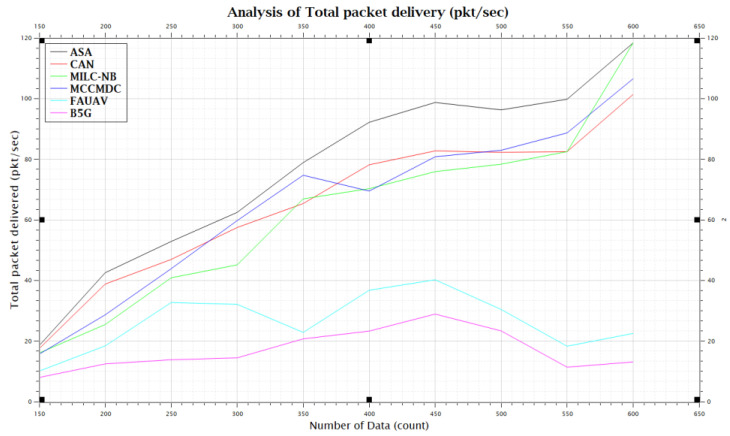
Packet delivery ratio before data obfuscation.

This section presents the simulation results showing the effectiveness of the proposed algorithm. This performance simulation is executed using CloudSim Plus, a framework for modelling and simulating extensible clouds. It is a good research tool that can manage the complications coming from simulated environments since it is a fully configurable tool that allows the expansion and formulation of rules in every software stack component. The proposed Averaged One-Dependence Estimators (AODE) and SELECT Applicable Only To Parallel Server (ASA) compare with the Beyond fifth Generation (B5G), Fully Automated Unmanned Aerial Vehicles (FAUAV) [2], Maximum Correlation Criterion, And Minimum Dependence Criterion (MCCMDC) [40], Multi Independent Latent Component Naive Bayes Classifier (MILC-NB) [3] and Correlation-Augmented Naïve Bayes (CAN) [28] Algorithm. The analyze results are then shown in Table 1 and Figure 4.

The database storage and retrieval issue are resolved by this research’s proposed Secure Cloud and Crowd Computing for Smart City Data Obfuscation. According to the results, the proposed method for data obfuscation increases total packet delivery by about 47.55%, CAN [28] delivery by about 50.55%, MILC-NB [3] delivery by about 52.73%, MCCMDC [38] delivery by about 55.80%, FAUAV [2] delivery by about 58.35%, and B5G [7] delivery by about 60.23% in comparison with the proposed algorithm.

**Figure 5:**
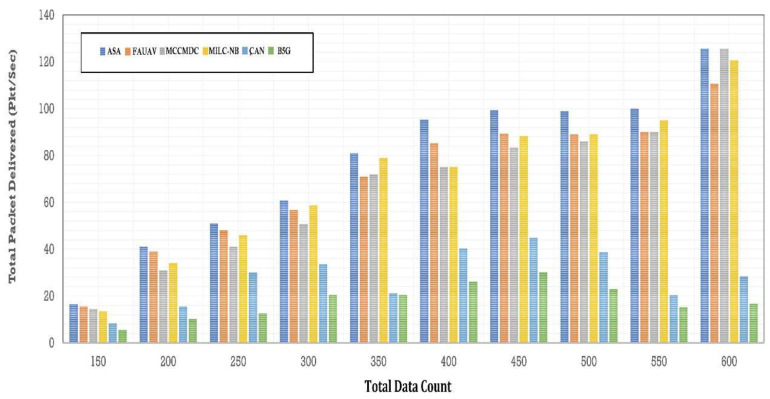
Packet delivery ratios before data obfuscation.

The ASA increases the total packet delivered by about CAN [28] in Figure 5. Packet delivery ratio after data obfuscation: MCCMDC [38] has 79.1%, FAUAV [2] has 83.7%, B5G [7] has 78.8%, and ASA has 81.3%, respectively.

**Figure 6:**
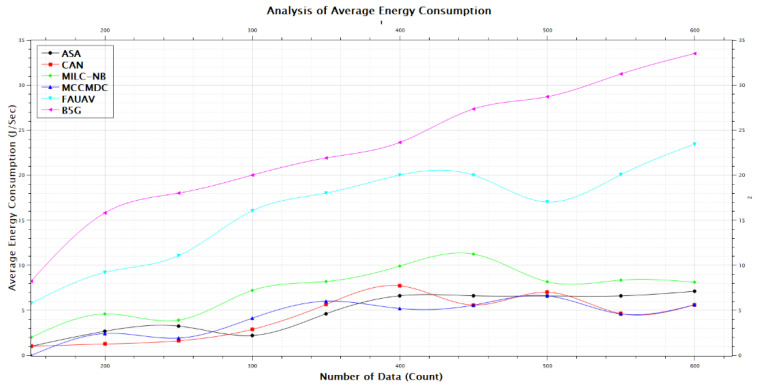
The average energy consumption before data obfuscation.

**Figure 7:**
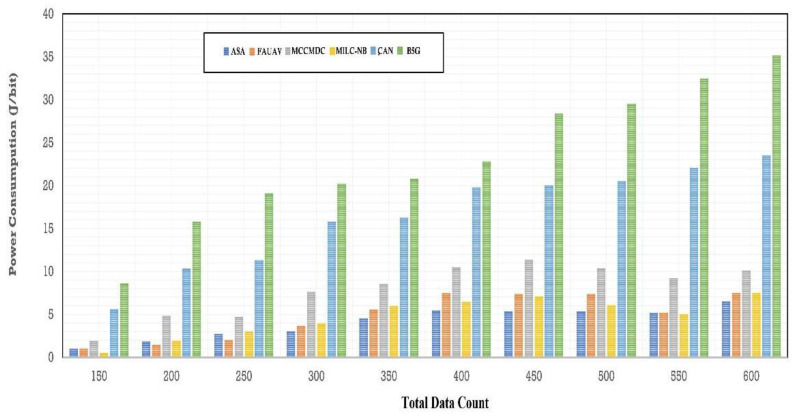
The power energy consumption after data obfuscation.

Table 2, Figure 6 and Figure 7 of this research deal with the obfuscation of data which renders sensitive information worthless to harmful actors by replacing it with data that seems to be actual production data. In this era, the Energy Consumption was calculated on the proposed Averaged One-Dependence Estimators (AODE) and SELECT Applicable Only to Parallel Server (ASA). This might be compared with the existing system of Energy Consumption. The Energy Consumption before data obfuscation is 1.18% lower than the CAN [28], 1.08% lower than the MILC-NB [3], 1.80% lower than the MCCMDC [38], 1.05% lower than the FAUAV [2], 4.02% lower than the B5G [7] algorithm, respectively. Meanwhile, the Average Energy Consumption after data obfuscation has 4.74%, decreased on CAN [28], 4.31%, decreased on MILC-NB [3], 7.18% decreased on MCCMDC [38], 4.21% decreased on FAUAV [2], 16.08% decreased on B5G [7] compared to the proposed Averaged One-Dependence Estimators (AODE) and SELECT Applicable Only to Parallel Server (ASA) algorithm.

**Figure 8:**
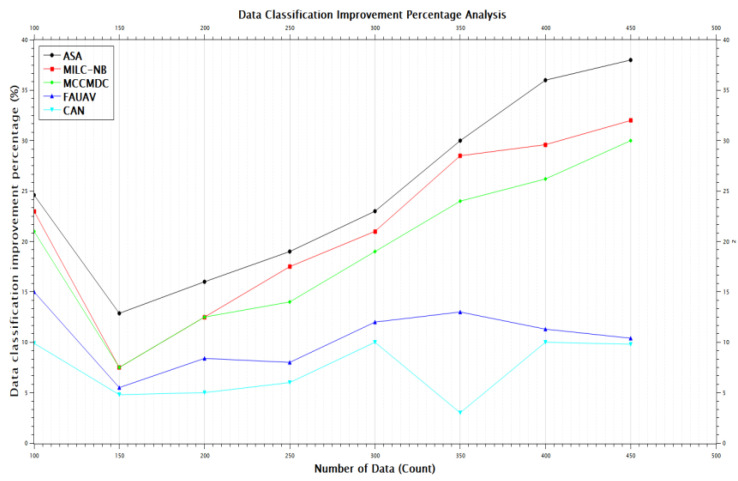
The improvement of Data classification improvement percentage for the ASA compared to other methods.

Table 3 and Figure 8 of this research: In terms of data classification improvement percentage in the proposed scenario, the proposed method has improved Data classification by about 3.48% compared with the MILC-NB algorithm, 5.66% compared with the MCCMDC algorithm, 14.48% compared with the FAUAV algorithm, and about 17.62% compared with the CAN approximately 69.34% compared with the Data classification improvement percentage Analysis on ASA algorithm.

**Figure 9:**
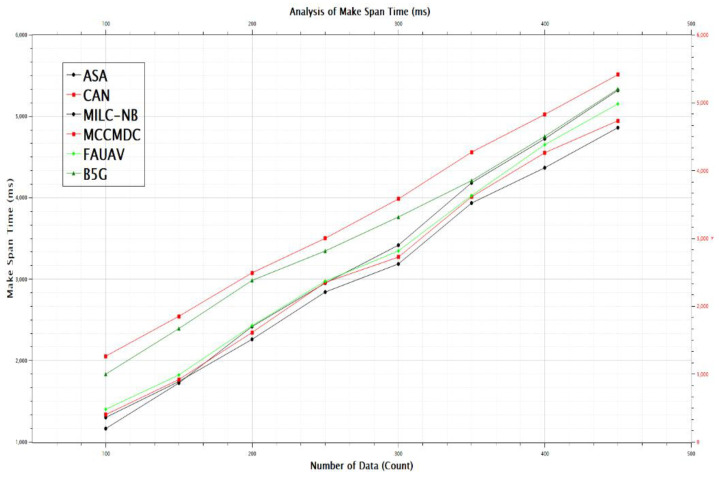
The Make span Before Cryptosystem Assumption.

**Figure 10:**
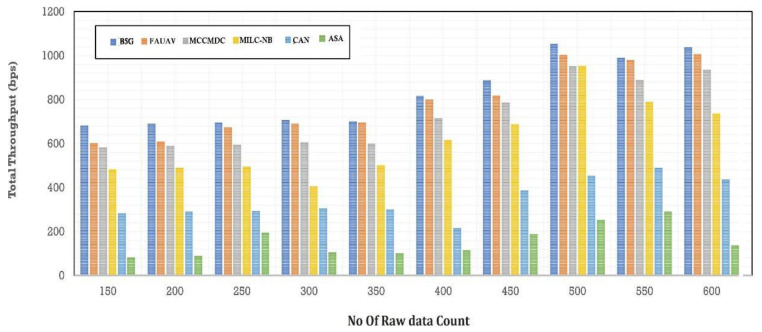
The total throughput after Cryptosystem Assumption.

The Analysis of Make Span Time (ms) before data obfuscation has been reduced by about CAN [28] 32.15%, MILC-NB [3] 33.20%, MCCMDC [38] 34.35%, FAUAV [2] 35.40%, B5G [7] 36.50%, and ASA 30.65%, which are shown in Table 4, Figure 9 and Figure 10. The overall throughput has dropped by about CAN [28] 1.27%, MILC-NB [3] 1.31%, MCCMDC [38] 1.36%, FAUAV [2] 1.41%, B5G [7] 1.44%, and ASA 1.21%, respectively, following Cryptosystem Assumption after data obfuscation.

**Figure 11:**
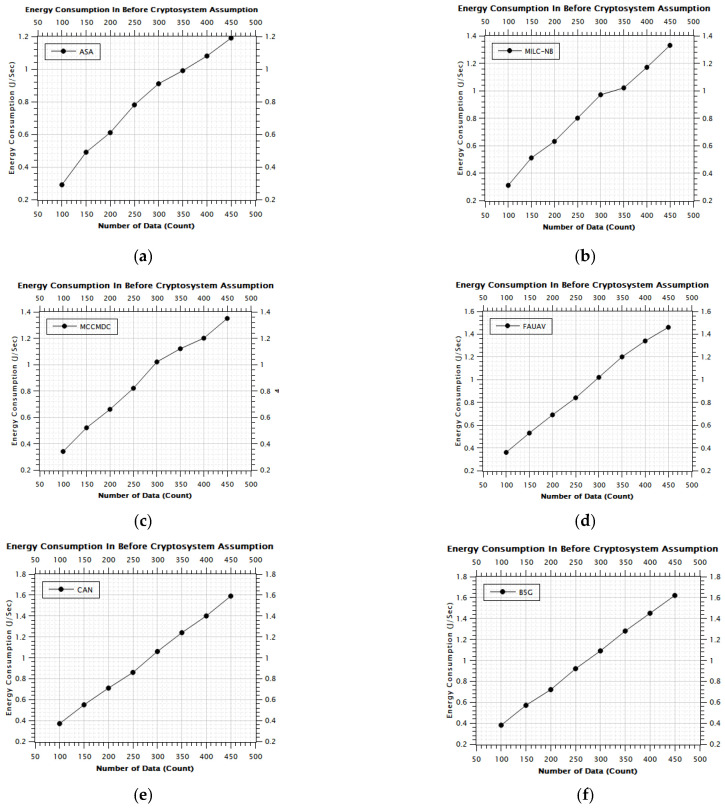
The energy consumption after cryptosystem assumption. (**a**) Energy Consumption In Before Cryptosystem (ASA), (**b**) Energy Consumption In Before Cryptosystem (MILC-NB), (**c**) Energy Consumption In Before Cryptosystem (MCCMDC), (**d**) Energy Consumption In Before Cryptosystem (FAUAV), (**e**) Energy Consumption In Before Cryptosystem (CAN), (**f**) Energy Consumption In Before Cryptosystem (B5G).

From Table 5, results in Analysis (Figure 11a–f) of Energy Consumption Before Cryptosystem Assumption show that the proposed method in Cryptosystem Assumption increases the Total packet delivered by about 79.25%, 84.25%, 87.88%, 93.00%, 97.25%, and 98.38% in comparison to MILC-NB, MCCMDC, FAUAV, CAN and B5G algorithm respectively. In terms of Energy Consumption improvement percentage in the proposed scenario, the proposed method has improved Energy Consumption by about 5.00% compared with the MILC-NB algorithm, 8.63% compared with the MCCMDC algorithm, 13.75% compared with the FAUAV algorithm, and about 18.00% compared with the CAN approximately 21.13% compared with the Data classification improvement percentage Analysis ASA algorithm.

**Figure 12:**
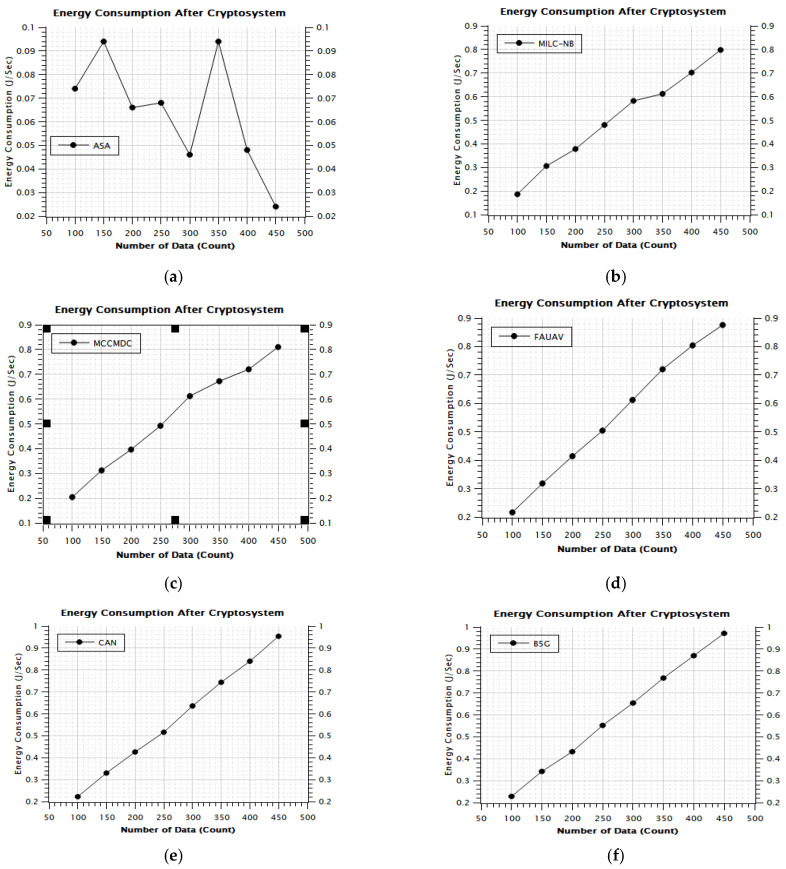
The energy consumption after cryptosystem assumption. (**a**) Energy Consumption After Cryptosystem (ASA), (**b**) Energy Consumption After Cryptosystem (MILC-NB), (**c**) Energy Consumption After Cryptosystem (MCCMDC), (**d**) Energy Consumption After Cryptosystem (FAUAV), (**e**) Energy Consumption After Cryptosystem (CAN), (**f**) Energy Consumption After Cryptosystem (B5G).

The proposed method, assuming the use of a Cryptosystem, increases the Total packet delivered by approximately CAN [28] 6.43%, MILC-NB [3] 50.55%, MCCMDC [38] 52.73%, FAUAV [2] 5.80%, B5G [7] 58.35%, and ASA 60.23% improved, according to Table 6’s results of the analysis (Figure 12a–f) of Energy Consumption assuming the use of a Cryptosystem. In terms of the proposed scenario’s Data classification improvement percentage, the proposed method outperformed the MILC-NB algorithm by about 6.43%, the MCCMDC algorithm by about 50.55%, the FAUAV algorithm by about 52.73%, the CAN algorithm by about 55.80%, and the Data classification improvement percentage Analysis ASA algorithm by about 60.23%.

## 5. Conclusions

This research work primarily focuses on data maintenance collected from IoT sensors in smart cities being managed by cloud computing. The three methods are data obfuscation, classification, and crowd computing for efficient data handling on the cloud using a cryptosystem. The introduced crowd computing is useful for data classification when using IoT sensors. The algorithm for collecting data from sensors improved data management with the help of hybrid data obfuscation. The AODE classifier helps to classify the data. The hybrid data obfuscation used data masking to maintain data management. SELECT-APSL was employed for data retrieving from the cloud. Finally, secure data was created in the presence of tracing data behaviour. The analysis results on energy consumption before and after cryptosystem use show that the proposed method increases total packet delivery by 79.25%, 84.25%, 87.88%, 93.00%, 97.25%, and 98.38% in comparison with MILC-NB, MCCMDC, FAUAV, CAN and B5G algorithm, respectively. The proposed method improved data classification by 6.43% compared with the MILC-NB algorithm, 50.55% compared with the MCCMDC algorithm, 52.73% compared with the FAUAV, 55.80% compared with CAN, and finally, 60.23% compared with the Data classification improvement percentage Analysis ASA algorithm. The analysis of makespan time (in milliseconds) after data obfuscation has decreased by 1.27%, 1.31%, 1.36%, 1.41%, 1.44%, and 1.21%, compared with CAN, MILC-NB, MCCMDC, FAUAV, and B5G algorithm, respectively.

## Figures and Tables

**Table 1:** Analysis of Total packet delivered (pkt/s).

No. of Data	Total Packet Delivered (PKT/sec)					
	ASA	CAN [13]	MILC-NB [10]	MCCMDC [26]	FAUAV [5]	B5G [4]
150	18.5	17.5	16.04	15.67	10.08	7.98
200	42.54	38.79	25.45	28.65	18.40	12.45
250	52.85	46.92	40.86	43.87	32.7	13.82
300	62.45	57.46	45.14	59.74	32.104	14.44
350	78.87	65.36	66.91	74.70	22.84	20.71
400	92.17	78.15	70.27	69.47	36.75	23.27
450	98.71	82.73	75.89	80.79	40.21	28.89
500	96.27	82.27	78.35	82.94	30.42	23.35
550	99.78	82.49	82.49	88.67	18.28	11.36
600	118.345	101.35	118.36	106.53	22.51	13.09

**Table 2:** Analysis of Average Energy Consumption.

No. of Data	Average Energy Consumption (J/sec)					
	ASA	CAN	MILC-NB	MCCMDC	FAUAV	B5G
150	1.023	1.024	2.005	0.001	5.782	8.246
200	2.682	1.264	4.602	2.430	9.213	15.825
250	3.257	1.623	3.921	1.923	11.078	18.023
300	2.213	2.891	7.218	4.132	16.057	20.021
350	4.621	5.652	8.210	6.020	18.025	21.925
400	6.623	7.732	9.924	5.213	20.023	23.651
450	6.623	5.592	11.253	5.582	20.023	27.365
500	6.603	7.017	8.183	6.612	17.032	28.721
550	6.613	4.643	8.359	4.603	20.062	31.254
600	7.126	5.621	8.129	5.621	23.457	33.521

**Table 3:** Data classification improvement percentage Analysis.

No. of Data	Data Classification Improvement Percentage (%)				
	ASA	MILC-NB	MCCMDC	FAUAV	CAN
100	24.6	23	21	15	9.9
150	12.87	7.5	7.5	5.5	4.8
200	16	12.5	12.5	8.4	5
250	19	17.5	14	8	6
300	23	21	19	12	10
350	30	28.5	24	13	3
400	36	29.6	26.2	11.3	10
450	38	32	30	10.4	9.8

**Table 4:** Analysis of Make Span Time (ms).

No. of Data	Make Span Time (ms)					
	B5G	CAN	MILC-NB	MCCMDC	FAUAV	ASA
100	1165	1265	1362	1403	1482	1000
150	1725	1853	1892	1921	1989	1672
200	2418	2493	2513	2614	2715	2381
250	2951	3002	3210	3351	3370	2815
300	3417	3582	3624	3725	3816	3316
350	4183	4271	4521	4612	4631	3852
400	4723	4826	5041	5262	5381	4504
450	5319	5412	5632	5734	5982	5201

**Table 5:** Analysis of Energy Consumption before Cryptosystem Assumption.

No. of Data	Energy Consumption before Cryptosystem					
	ASA	MILC-NB	MCCMDC	FAUAV	CAN	B5G
100	0.29	0.31	0.34	0.36	0.37	0.38
150	0.49	0.51	0.52	0.53	0.55	0.57
200	0.61	0.63	0.66	0.69	0.71	0.72
250	0.78	0.8	0.82	0.84	0.86	0.92
300	0.91	0.97	1.02	1.02	1.06	1.09
350	0.99	1.02	1.12	1.2	1.24	1.28
400	1.08	1.17	1.2	1.34	1.4	1.45
450	1.19	1.33	1.35	1.46	1.59	1.62

**Table 6:** Analysis of Energy Consumption after Cryptosystem Assumption.

No. of Data	Energy Consumption after Cryptosystem					
	ASA	MILC-NB	MCCMDC	FAUAV	CAN	B5G
100	0.074	0.186	0.204	0.216	0.222	0.228
150	0.094	0.306	0.312	0.318	0.33	0.342
200	0.066	0.378	0.396	0.414	0.426	0.432
250	0.068	0.48	0.492	0.504	0.516	0.552
300	0.046	0.582	0.612	0.612	0.636	0.654
350	0.094	0.612	0.672	0.72	0.744	0.768
400	0.048	0.702	0.72	0.804	0.84	0.87
450	0.024	0.798	0.81	0.876	0.954	0.972

## Data Availability

The data presented in this study are available on request from the corresponding author.
